# Gene expression profiling meta-analysis reveals novel gene signatures and pathways shared between tuberculosis and rheumatoid arthritis

**DOI:** 10.1371/journal.pone.0213470

**Published:** 2019-03-07

**Authors:** M. T. Badr, G. Häcker

**Affiliations:** 1 Institute of Medical Microbiology and Hygiene, Medical Center—University of Freiburg, Faculty of Medicine, Freiburg, Germany; 2 BIOSS Centre for Biological Signaling Studies, University of Freiburg, Freiburg, Germany; Universiti Sains Malaysia, MALAYSIA

## Abstract

Tuberculosis (TB) is among the leading causes of death by infectious diseases. An epidemiological association between *Mycobacterium tuberculosis* infection and autoimmune diseases like rheumatoid arthritis (RA) has been reported but it remains unclear if there is a causal relationship, and if so, which molecular pathways and regulatory mechanisms contribute to it. Here we used a computational biology approach by global gene expression meta-analysis to identify candidate genes and pathways that may link TB and RA. Data were collected from public expression databases such as NCBI GEO. Studies were selected that analyzed mRNA-expression in whole blood or blood cell populations in human case control studies at comparable conditions. Six TB and RA datasets (41 active TB patients, 33 RA patients, and 67 healthy controls) were included in the downstream analysis. This approach allowed the identification of deregulated genes that had not been identified in the single analysis of TB or RA patients and that were co-regulated in TB and RA patients compared to healthy subjects. The genes encoding TLR5, TNFSF10/TRAIL, PPP1R16B/TIMAP, SIAH1, PIK3IP1, and IL17RA were among the genes that were most significantly deregulated in TB and RA. Pathway enrichment analysis revealed ‘T cell receptor signaling pathway’, ‘Toll-like receptor signaling pathway,’ and ‘virus defense related pathways’ among the pathways most strongly associated with both diseases. The identification of a common gene signature and pathways substantiates the observation of an epidemiological association of TB and RA and provides clues on the mechanistic basis of this association. Newly identified genes may be a basis for future functional and epidemiological studies.

## Introduction

Tuberculosis (TB) is an infectious disease caused predominantly by *Mycobacterium tuberculosis* (Mtb). With estimated 10.4 million active cases and 1.3 million TB-related deaths per year, TB is one of the most important infections worldwide [1]. In healthy subjects, contact with *M*. *tuberculosis* results mostly in chronic, latent infection without clinical symptoms, but a small number of patients move on to develop active TB. Other than the lack of T cell function, which drives active TB especially in HIV-patients, the factors determining predisposition to the progression to active TB are not well understood although several clinical conditions predispose to active TB [2,3]. TB has many extrapulmonary manifestations, with bone and joint involvement being the most common (10–11% of extrapulmonary TB) [4].

Rheumatoid arthritis (RA) is a chronic systemic inflammatory autoimmune disorder that mainly causes symptoms in the synovial joints such as swelling, pain, and stiffness. RA is estimated to affect approximately 0.5% to 1% of the world’s population. Extra-articular manifestations such as subcutaneous nodules, pericarditis, pulmonary effusion and arteritis are common in RA. The main pathological pathways and underlying mechanisms that initiate and lead to the development of RA remain undetermined [5].

Infections with several agents including Mtb have been repeatedly found to be associated with a wide variety of conditions and syndromes [6] of immune deregulation such as sarcoidosis, psoriasis, Sjögren’s syndrome, systemic lupus erythematosus, and rheumatoid arthritis [7–10]. TB-patients are specifically known to produce antibodies that are also found in autoimmune syndromes such as anticyclic citrullinated peptide (anti-CCP) and anti-arginine-containing peptide (anti-CAP) [11,12].

(Active) TB and RA are different conditions with different pathogenesis. They however share aspects of chronic immune activation and the concept of immune deviation. Since only a minority of patients develop active TB it may be argued that a specific form of immune reactivity is required. In RA, although the trigger is unknown, the immune system is activated in an unwarranted fashion. There is some evidence of similar immunological activity in both conditions. RA-patients often show a good response to immunosuppressive drugs, notably to blockade of tumor necrosis factor (TNF) signaling. Such blockade of the RA immune activity can drive the progression of latent to active TB, suggesting that mechanisms that drive RA play a role in containing TB [13–15].

Recent studies have further shown that infection with Mtb may induce or at least aggravate arthritis. In an arthritis model, mice treated with collagen emulsion plus killed *M*. *tuberculosis* showed significantly elevated arthritis scores, while control mice treated with the collagen emulsion alone did not develop arthritis [16]. Furthermore, a significant osteoclast presence in the subchondral bones and increased serum-levels of IL-6 were seen in Mtb-infected mice. A contribution from Toll-like receptor 2 (TLR2) has been described, as TLR2 deficient mice showed significantly less disease-severity in the same model, and TLR2 has been shown in other studies to regulate various invasive mechanisms in RA [17]. Previous population-based studies have further found an unexpectedly high prevalence of TB in RA patients, an association that was even stronger than the established association with other comorbidities such as kidney disease, diabetes, and hypertension [18,19]. Despite this epidemiological and mechanistically suggestive evidence of a link between TB and RA, there are no mechanistic explanations for this association.

Global gene expression analysis is a well-established way of characterizing complex cellular responses. Novel molecular pathways have been identified in various conditions using this approach [20]. The many publicly available data permit pooling gene expression datasets and to increase sensitivity by increasing the number of data points. This strategy has already been used to identify gene signatures and pathways that are co-regulated in a number of autoimmune conditions [21]. Similarly, a gene-expression meta-analysis has been successful in identifying novel genes and pathways deregulated in active TB [22]. We here use this in silico approach to test the hypothesis that immune responses common to both TB and RA exist that are either the result of or even perhaps a mechanistic basis for the association between the two types of disease. We used publicly available microarray datasets to investigate gene co-expression patterns of human blood cells in TB and RA patients. To the best of our knowledge, this is the first study to investigate the shared molecular pathways between RA and TB using this approach.

## Methods

### Data collection

Collection of the meta-analysis data was carried out by searching public expression databases (NCBI GEO and Array Express; however only GEO data were eventually included). We used following search terms: rheumatoid arthritis, RA, tuberculosis, TB, *Mycobacterium tuberculosis*) and the filters (organism (Homo sapiens)), study type (expression profiling by array), entry type (Dataset/Series)). Initially 787 entries were recovered. Duplicates and irrelevant studies were excluded, and 34 studies remained. These studies were further refined using the inclusion criteria (below) to reach the 6 final studies included in our analysis.

We included only studies that had analyzed gene expression in whole blood, PBMC or blood cell components but excluded studies using other tissues such as synovial fluid, chondrocytes or lung tissue to ensure comparable gene expression and to remove potential bias through tissue specific gene expression. Only samples from untreated TB or RA were included. Studies investigating patients with latent TB were also excluded. In one case (Dataset GSE62525) we were unable to annotate the data properly; this was also excluded. The database-search followed the Preferred Reporting Items of Systematic reviews and Meta-Analyses (PRISMA) statement and is documented in the PRISMA Flow Diagram (S1 File) [23].

After a thorough search and excluding datasets as specified above, two datasets for RA (GSE15573 and GSE4588) and 4 TB datasets (GSE54992, GSE65517, GSE19435, and GSE19444) [24–27] were selected for further analysis. A total of 141 samples were considered for downstream analysis, containing data from 41 TB patients, 33 RA patients, and 67 healthy controls. The R programming language was used for initial processing and analysis of the datasets. The datasets were downloaded from the NCBI GEO database using the GEOquery R package [28]. As the original CEL files for the dataset GSE4588 were unavailable, the deposited gene expression matrix was directly retrieved from the NCBI GEO database using the GEOquery R package and processed as previously described [21]. After including it in our meta-analysis pipeline and cross study normalization we investigated the effect of batch normalization by principle component analysis. As no bias was detected this dataset was considered suitable for analysis. For each study we extracted the GEO accession number, platform, sample type and gene expression data. The microarray chip identifiers were transformed to other suitable Gene IDs including Entrez Gene identifiers for downstream analysis. Datasets were merged after annotation with the Entrez Gene identifiers. A suitable identification condition for each sample (case or control) and class (TB, RA, and healthy control) were assigned, and further analysis was carried out with the web-based tool NetworkAnalyst [29,30].

### Data processing

Normalization by Log2 transformation with autoscaling to each dataset was performed. Each dataset was then visually inspected using PCA plots to insure the absence of outliers. The individual analysis of each dataset was carried out using the Benjamini–Hochberg's False Discovery Rate (FDR) [31] with cut-off p-values of <0.05. To adjust for the batch effect between the different datasets we used the ComBat batch effect method through the INMEX tool [32]. For detecting the significantly deregulated genes between cases (TB or RA) and controls, the effect size method was used. This approach offers two models for the analysis, the fixed and random effects models (FEM and REM). The most suitable model can be determined by measuring the statistical heterogeneity estimation by Cochran’s Q tests. Based on the Cochran’s Q test we settled on the REM, which usually gives more conservative results, by extracting fewer DEGs but with more confidence. We set a discovery significant value of <0.01 using the REM to discover the most significant DEGs in our downstream analysis. A heatmap of DEGs was created using the visual inspection tools of NetworkAnalyst and clustered using single linkage method.

### Hub genes network analysis

NetworkAnalyst was implemented to generate a protein-protein interaction (PPI) network by integrating the innateDB interactome database [33]. With the original seed of 304 deregulated genes a first-order PPI network was generated with 3,433 nodes representing the proteins and 6,851 edges representing the interaction between these proteins. For better visualization of the network and focusing on key connections a zero-order PPI network was created with 57 nodes connected with 80 edges.

### Integrative pathway analysis

Identifying the overrepresented biological terms in the deregulated genes dataset was carried out using DAVID (Database for Annotation, Visualization, and Integrated Discovery) [34]. The analysis was performed by uploading a list of the Entrez Gene identifiers comparing the genes on that list to all human genes in the genome. The Functional Annotation Chart and Clustering program was used in accordance with the developer’s protocol [34]. Default settings of the functional annotation chart and clustering tool were used, and Fisher's exact tests were used to calculate p-values. To rank the cluster of terms based on their biological significance a group enrichment score is used, meaning that the top ranked groups would have higher p-value for their member genes. To better visualize and explore the enriched pathways and biological terms related to our DEGs, we redid the analysis using the ClueGo v2.5.0 [35,36] tool, a visualization plug-in implemented in the Cytoscape v3.6.0 environment. We included the EBI Gene Ontology and GO Annotations QuickGO [37,38], KEGG [39], and Reactome pathway databases [40,41] for our analysis and implemented the Go term fusion option to exclude redundancy, with enrichment (left-sided) hypergeometric distribution tests. The leading biological terms were ranked based on their significance with a p-value significance level of ≤0.05, followed by the Bonferroni adjustment for the terms and the groups with Kappa-statistics score threshold set to 0.3.

### Transcription factor analysis

To detect the overrepresented transcription factor binding sites (TFBS) from DEGs, the transcription factor discovery module in NetworkAnalyst was implemented, and processing was carried out against both the JASPAR and ENCODE databases. To discover further possible connections between DEGs and transcription factors, we repeated the analysis using the EnrichR tool, which computes enrichment through 35 different gene-set libraries [42,43]. We detected the binding motif sites in our gene list using the position weight matrices (PWMs) analysis from TRANSFAC and JASPAR. The PWMs from TRANSFAC and JASPAR were used to scan the promoters of all human genes in the region between −2,000 and +500 from the transcription factor start site (TSS).

## Results

### Meta-analysis data selection and preparation pipeline

From the initial datasets acquired by searching public databases, six matched our predetermined inclusion criteria (see methods). A detailed pipeline of inclusion and analysis workflow can be found in S1 Fig. The six datasets included in the further analysis contained samples from 41 patients with active TB, 33 RA patients, and 67 healthy controls (141 samples in total). The data summary of the included datasets and samples can be found in S1 and S2 Tables.

### Data acquisition and normalization

The individual dataset gene expression normalization was carried out using the NetworkAnalyst log2 transformation function, followed by autoscaling. The individual datasets were inspected with PCA plots before and after normalization, and PCA plots of gene expression data of the 6 datasets before and after normalization are shown in S2 and S3 Figs. No major differences were seen that could be attributed to differences in dataset platforms or conditions and that could have introduced a bias.

### Identification of a common gene expression signature of TB and RA

**Fig 1:**
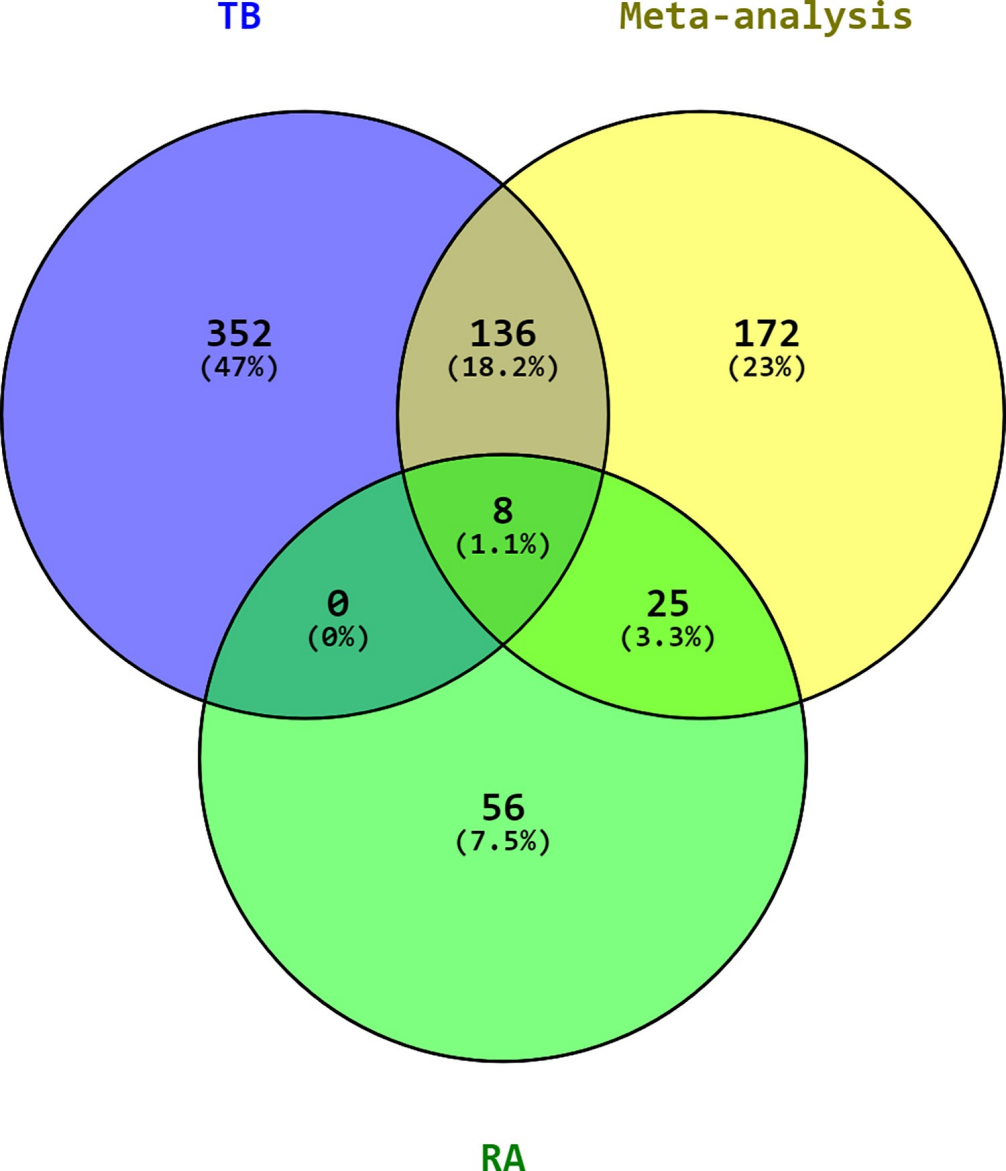
Venn diagram of DEGs. In comparison to the individual meta-analysis of the Tuberculosis (TB) and rheumatoid arthritis (RA) datasets, the combined meta-analysis of both diseases shows many DEGs (172) that only were significantly different following this approach. Loss of genes that were only significant in their respective disease datasets (genes that play no role common to both conditions) is also expected. Data sets were analyzed with the same parameters in NetworkAnalyst, and genes with a p-value < 0.01 were considered significant.

Based on the results of Cochran’s Q test (S4 Fig), the REM in NetworkAnalyst was used. Using REM, 341 genes were identified as significantly differentially expressed between the TB and RA datasets versus healthy controls (p<0.01 in the REM). A list of the 50 most significantly up- or downregulated genes is shown in S3 Table. A main hypothesis of this meta-analysis was that through the inclusion of datasets from both TB and RA, DRGs would be identified that had not been significantly regulated in the analysis of individual datasets and in the analysis of only one condition. As illustrated in the Venn diagram (Fig 1), this was indeed the case. A total of 172 DEGs were found to be only significantly deregulated in the meta-analysis but not in the analysis of individual conditions. Further, 408 genes were identified that were only significant in the individual disease analysis but not the meta-analysis.

**Fig 2:**
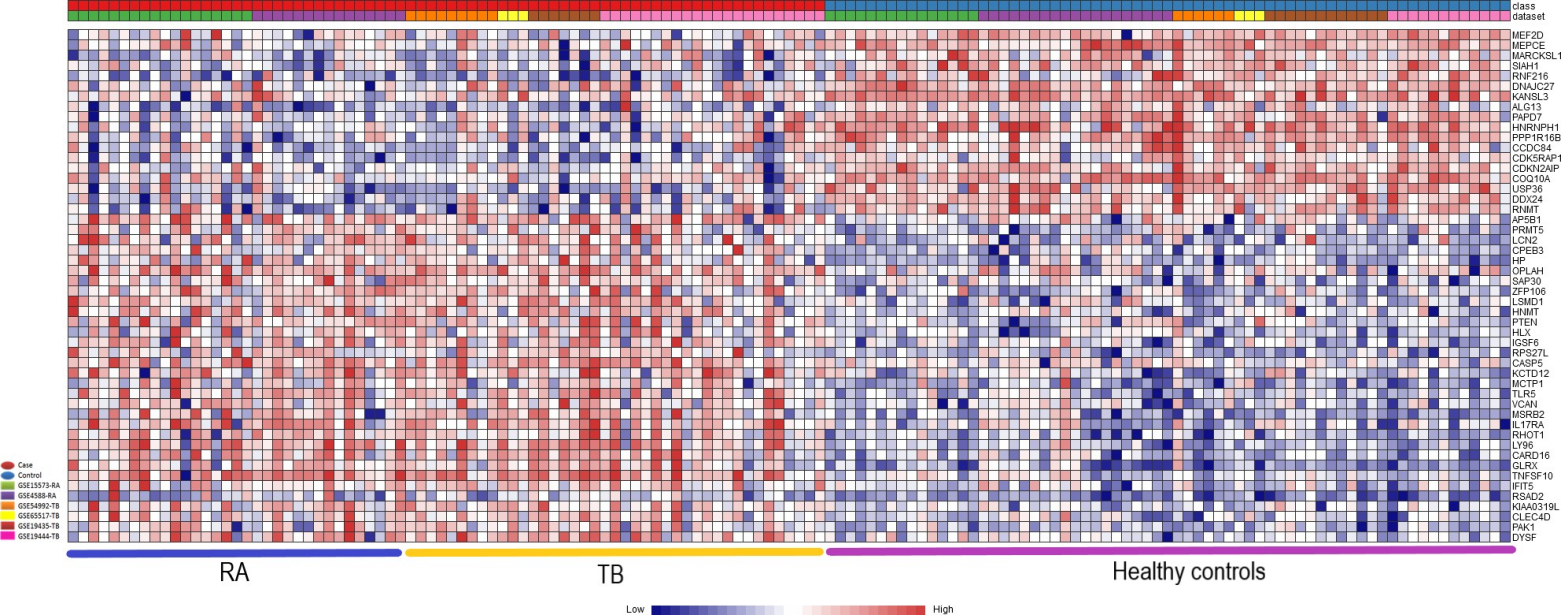
Heatmap of most significantly differentially expressed genes. Heatmap showing the relative expression of the 50 most significantly differentially expressed genes (DEGs) of the 341 significant DEGs identified through the meta-analysis, where 205 genes were co-up-regulated, and 136 genes were co-down-regulated (TB and RA versus control). The heatmap indicates the normalized expression value of each DEG in the individual samples, and genes were clustered based on their condition (cases vs controls) and their original datasets. The heatmap was created by the visualization module in NetworkAnalyst, and genes with p-value < 0.01 in the Random Effect Model analysis were considered significant.

Particularly striking was the highly significant upregulation of the genes encoding Toll-like receptor 5 (TLR5) (combined ES of 1.467 and adjusted p-value of 2.47E-09) and death receptor ligand TNFSF10/TRAIL (combined ES of 2.0036 and adjusted p-value of 4.86E-09), as well as the downregulation of protein phosphatase 1 regulatory subunit 16B PPP1R16B/TIMAP (combined ES of 1,3586 and adjusted p-value of 3,34E-07) and the E3 ubiquitin protein ligase 1 (SIAH1) (combined ES of 1,055 and adjusted p-value of 1,65E-05) in both conditions. In addition, numerous immune response regulating genes were significantly deregulated only in the meta-analysis, including DDX58 and CLEC7A. A complete list of the differentially regulated genes with the highest significance is shown in S3 Table. A heatmap of the most highly differentially regulated genes is shown in Fig 2.

### Hub genes network analysis

**Fig 3:**
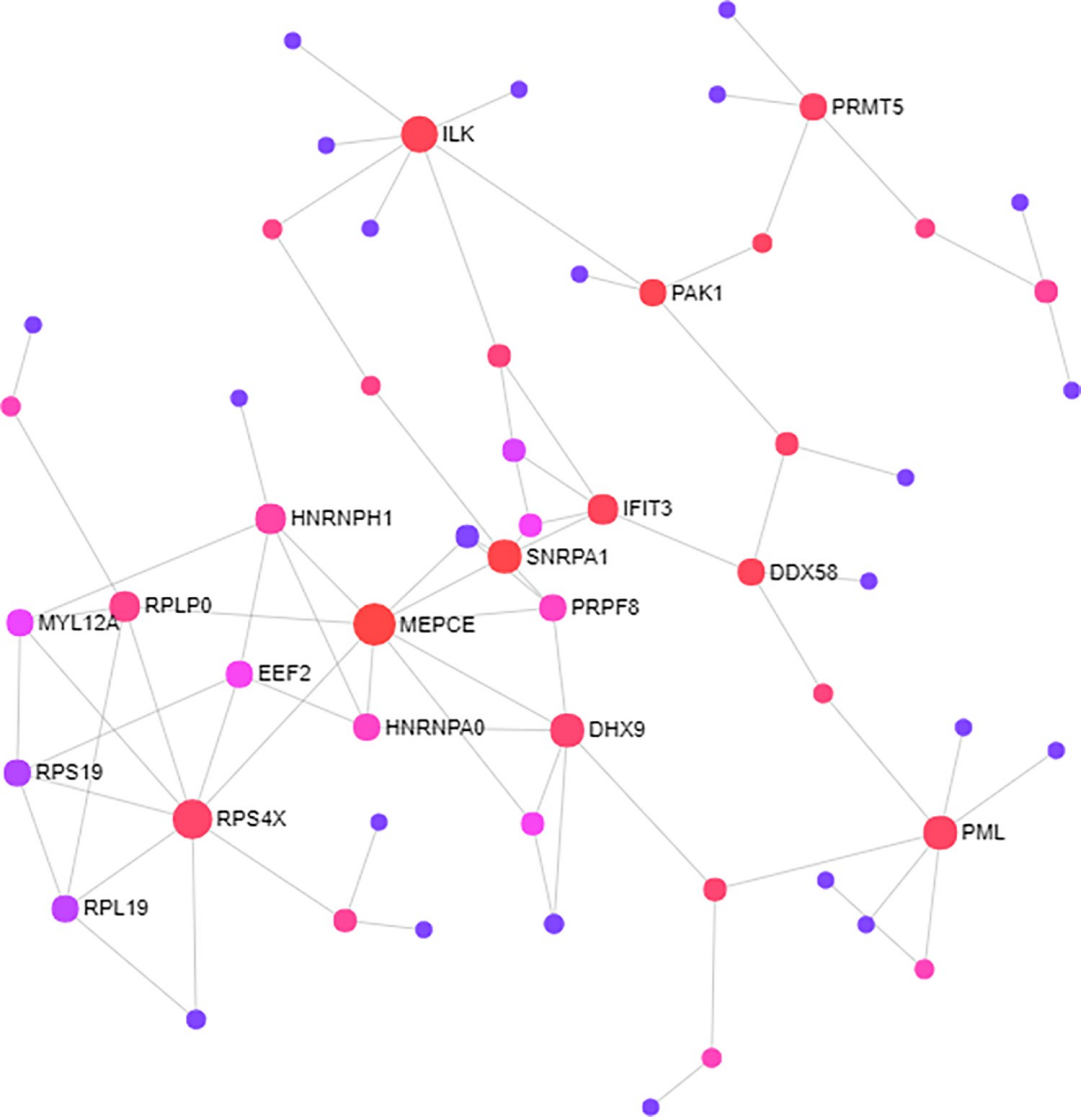
Network analysis of the most highly deregulated genes. Shared differentially expressed genes (DEGs) (TB and RA versus controls) were integrated in NetworkAnalyst tools to visualize gene interactions. A ‘zero order’ interaction network with 57 nodes was used. The most highly ranked nodes across the dataset based on network topology measures were MEPCE (betweenness centrality = 662.8; degree = 9), and RPS4X (betweenness centrality = 294.76; degree = 8).

To extract more biologically relevant information, we performed a network-based analysis. This analysis identified key hub genes among the most highly deregulated genes (Fig 3). MEPCE with the combined ES of -1.0558 and adjusted p-value of 1.65E-05 and RPS4X with the combined ES of -0.86739 and adjusted p-value of 0.00045462 were found to be among the most highly ranked hub genes among the downregulated DEGs, while ILK (combined ES of 0.96394 and adjusted p-value of 0.0061503) and PML (combined ES of 0.74222 and adjusted p-value of 0.003701) were among the most highly ranked hub genes in the overexpressed DEGs.

### Identification of overrepresented biological pathways and gene ontology terms

Pathway enrichment analysis was first performed using the KEGG based pathway enrichment identification module in NetworkAnalyst using the 341 genes identified as significantly deregulated (S4 Table). As expected, infection related pathways and host immune defense pathways such as the Toll-like receptor signaling pathway were found among the most highly enriched genes. Further pathways were found that may be linked to the triggering of autoimmunity in RA, such as osteoclast differentiation and T cell receptor signaling pathways. We further conducted enrichment analysis using the functional annotation chart tool of DAVID (Table 1) and the functional annotation clustering tool, which permits clustering of biologically related groups/terms with similar annotations and genes. The two most strongly enriched pathway clusters are the innate immunity related pathway with an enriched cluster score of 7.415 and antiviral defense pathway cluster with an enriched score of 3.60.

**Table 1:** Overrepresented biological pathways and gene ontology terms.

Category	Term	p-value	Fold Enrichment
**UP_KEYWORDS**	Innate immunity	7,65E-11	5,549870226
**UP_KEYWORDS**	Immunity	5,34E-09	3,621290323
**GOTERM_CC_DIRECT**	GO:0005829~cytosol	1,70E-05	1,531793284
**GOTERM_CC_DIRECT**	GO:0005739~mitochondrion	1,99E-05	1,949938709
**GOTERM_MF_DIRECT**	GO:0005515~protein binding	2,06E-05	1,22680094
**UP_KEYWORDS**	Antiviral defense	2,30E-05	5,723303671
**UP_KEYWORDS**	RNA-binding	4,84E-05	2,450497211
**GOTERM_BP_DIRECT**	GO:0045087~innate immune response	1,39E-04	2,65396252
**GOTERM_BP_DIRECT**	GO:0009615~response to virus	1,92E-04	4,940276552
**UP_KEYWORDS**	Acetylation	2,34E-04	1,463040775
**GOTERM_BP_DIRECT**	GO:0051607~defense response to virus	2,38E-04	3,952221242
**GOTERM_CC_DIRECT**	GO:0005654~nucleoplasm	2,63E-04	1,49969752
**UP_KEYWORDS**	Cytoplasm	3,69E-04	1,353472297
**UP_KEYWORDS**	Phosphoprotein	4,03E-04	1,229640177
**UP_KEYWORDS**	Ribonucleoprotein	4,37E-04	3,058522232
**GOTERM_BP_DIRECT**	GO:0006364~rRNA processing	6,15E-04	3,301212836
**GOTERM_BP_DIRECT**	GO:0045071~negative regulation of viral genome replication	7,81E-04	8,151456311
**GOTERM_MF_DIRECT**	GO:0003727~single-stranded RNA binding	0,001036942	7,672600561
**GOTERM_BP_DIRECT**	GO:0060337~type I interferon signaling pathway	0,001113152	5,943770227
**UP_KEYWORDS**	Nucleus	0,001158284	1,300552398
**UP_KEYWORDS**	Transferase	0,001235365	1,625481605
**GOTERM_MF_DIRECT**	GO:0044822~poly(A) RNA binding	0,001330009	1,753348932
**UP_SEQ_FEATURE**	domain:PARP catalytic	0,002704252	13,92538608
**GOTERM_CC_DIRECT**	GO:0030529~intracellular ribonucleoprotein complex	0,002903681	3,73374613
**INTERPRO**	IPR012317:Poly(ADP-ribose) polymerase, catalytic domain	0,003148181	13,19282033
**UP_KEYWORDS**	Apoptosis	0,003655372	2,139443909
**GOTERM_MF_DIRECT**	GO:0019901~protein kinase binding	0,003734618	2,339871093
**GOTERM_MF_DIRECT**	GO:0004386~helicase activity	0,00445735	4,528338762
**GOTERM_MF_DIRECT**	GO:0003725~double-stranded RNA binding	0,004920079	5,408554494
**GOTERM_MF_DIRECT**	GO:0003723~RNA binding	0,005399115	2,01049253
**UP_SEQ_FEATURE**	mutagenesis site	0,006926513	1,431626296
**UP_KEYWORDS**	Atherosclerosis	0,007133992	22,63306452
**GOTERM_BP_DIRECT**	GO:0070207~protein homotrimerization	0,007267988	9,880553104
**INTERPRO**	IPR011545:DNA/RNA helicase, DEAD/DEAH box type, N-terminal	0,007657263	4,875607513
**UP_SEQ_FEATURE**	binding site:S-adenosyl-L-methionine; via carbonyl oxygen	0,009246675	9,105060132
**GOTERM_CC_DIRECT**	GO:0036464~cytoplasmic ribonucleoprotein granule	0,009424189	9,027368421

A list of the biological terms enriched with the highest significance (analyzed using the Functional Annotation chart module in DAVID). The p-values were calculated using a modified Fisher Exact test for gene-enrichment analysis and table summarize terms with p-value < 0.01.

**Fig 4:**
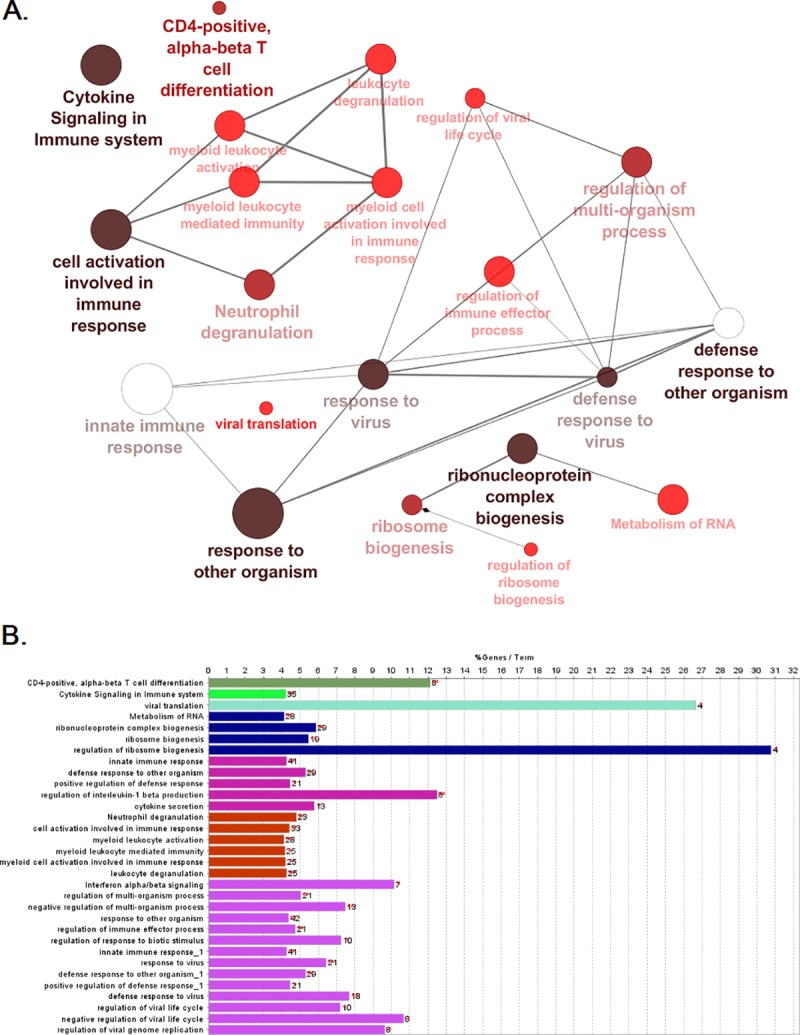
Over-represented biological pathways and gene ontology terms. Gene ontology and pathway enrichment analysis were conducted using the ClueGO plugin in Cytoscape. **A** the most significant terms where the node size correlates to the term enrichment significance. Pathways related to innate immunity activation and pathogen defense mechanisms appear as the most significant terms in our analysis. **B** the same most highly enriched terms shown as bars, where the bars represent the number of genes associated to this term, and the percentage of genes per term is shown as bar label.

To visualize the enriched pathways and biological terms in a nonredundant manner we fed the identified DEGs to the ClueGO (35) tool on the Cytoscope program. This method identified cytokine signaling in immune response, viral translation, CD4-positive αβ-T cell differentiation, and innate immune response among the most significant terms. A network showing the most highly enriched pathways and terms from this analysis is shown in Fig 4. The GO/pathway terms match to a great degree with the results obtained with the other tools such as DAVID or NetworkAnalyst.

### Analysis of transcription factors

We searched for overrepresented transcription factor binding sites (TFBS) in the identified DEGs, using the TF exploration module in NetworkAnalyst. We scanned the regions around these DEGs with all core vertebrate transcription factor binding profiles present in the JASPAR and ENCODE databases. A network of transcription factors in the JASPAR analysis is shown in S5 Fig. We further used the EnrichR web-based tool to elucidate other possible regulatory mechanisms that may affect these target genes through the detection of binding motif sites in the gene list using PWMs from TRANSFAC and JASPAR (Table 2).

**Table 2:** Significant transcription factor binding sites associated with DRGs.

Index	Name	P-value	Adjusted p-value	Z-score	Combined score
**1**	SOX10 (human)	0.0001388	0.04219	-2.03	18.03
**2**	TEAD1 (human)	0.0005657	0.05334	-1.74	12.99
**3**	APEX1 (human)	0.0006451	0.05334	-1.77	12.98
**4**	RARA (human)	0.0007018	0.05334	-1.74	12.66
**5**	FOS (human)	0.004997	0.3038	-1.72	9.12
**6**	NR1H2 (human)	0.008450	0.4281	-1.87	8.91
**7**	CEBPE (human)	0.02180	0.5956	-1.89	7.24
**8**	JUN (human)	0.01382	0.5956	-1.56	6.67
**9**	LOC135440 (human)	0.02634	0.5956	-1.78	6.47
**10**	CBX5 (human)	0.03050	0.5956	-1.83	6.40

Binding motifs were detected at the gene promoter using Enrichr Tool through scanning the TRANSFAC and JASPAR databases. The TRANSFAC and JASPAR Position weight Matrices (PWM) table list the most highly significant 10 transcription factors. The combined score is calculated by taking the log of the p-value from the Fisher exact test and multiplying that by the Z-score of the deviation from the expected rank.

## Discussion

These results illustrate the usefulness of global gene expression meta-analysis to extract information that would not be visible in the analysis of individual datasets. Not only does it provide a higher sensitivity for the combination of independent sets of similar patient-cohorts, for instance, when several studies of a similar nature are analyzed together, but when combining datasets from different conditions this approach also opens the possibility of identifying gene sets that are similarly regulated in separate conditions. We here used this approach to identify DRGs, pathways, and regulatory networks that are shared between the two conditions of TB and RA.

In this approach, 172 genes were found as differentially regulated in the disease cohorts only through the gene expression meta-analysis, besides many potentially shared pathways between RA and TB that can shed light on the mechanisms of TB triggered autoimmunity. Innate immunity pathways and antiviral defense pathways were identified as the most highly significantly enriched pathways. This may not be surprising *per se*, but it supports functional studies and provides the basis for in-depth analyses.

One of the earliest events that can be detected in RA-development is an innate immune response and the activation of various antigen-presenting cells such as dendritic cells and macrophages [44,45]. The host reaction against Mtb begins with the recognition of pathogen‐associated molecular patterns (PAMPs) by various pattern recognition receptors such as C-type lectin receptors [46], Toll‐like receptors (the C-type lectin receptor CLEC4D and TLR5/8 were found to be upregulated in our meta-analysis, see S2 Table), and the reaction of macrophages and dendritic cells (DC) [47]. It has also been long known that infection with Mtb leads to upregulation of various TLRs, in turn leading to the activation of proinflammatory signals and the production of cytokines [48]. TLR5, which is the most significantly upregulated gene in this analysis, is a member of the TLR family, best known for its binding to bacterial flagellin [49,50]. A mycobacterial ligand of TLR5 is not known. However, TLR5 has far-reaching immunoregulatory properties such as stimulation of IL17-production by dendritic cells in different tissues as the lungs, spleen and mucosa and the modulation of TLR2 and TLR4 antimicrobial response [51]. TLR5 can determine vaccine efficiency [52] and the composition of the microbiome [53]. Intriguingly, TLR5 has been identified as a mediator of osteoclast differentiation and bone loss [54], as well as of myeloid cell infiltration in RA [55]. TLR5-regulation may therefore contribute to the clinical association of RA and TB.

Key players in RA are B cells [56]. B-cell activating factor (TNFSF13B/BAFF), upregulated in the datasets analyzed here, is an important factor of humoral immunity and has been implicated in the overproduction of antibodies in RA [57–59]. In terms of signaling, BAFF activates the noncanonical NF-κB pathway [60], which has implications on B cell survival, maturation and T cell adhesion [61]. BAFF may therefore also be a factor that facilitates transition between TB and RA. All of these upstream immune signals through TLRs, DCs, T cells or B cells can lead to the downstream activation of fibroblast and osteoclasts, which invade the synovial membrane of RA patients and play a prominent role in the mediation of synovial inflammation and bone destruction [45,62].

In our analysis we also saw many genes that regulate cell death (necroptosis or pyroptosis) pathways, such as TRAIL (tumor necrosis factor ligand superfamily member 10; also involved in apoptosis), MLKL (mixed lineage kinase domain-like), EIF2AK2 and caspase-1/5 (all up-regulated). The role of programmed cell death during Mtb-infection by necroptosis, pyroptosis or apoptosis is still somewhat controversial with various studies suggesting (for different cell types) an enhancement or attenuation of cell death during TB infection [63]. A possible explanation to this complex picture is that Mtb may inhibit apoptotic/necroptotic signals in immune cells especially macrophages during initial stages of the disease to allow for replication, and then in more advanced stages induce cell death through MLKL-dependent necroptosis or apoptosis to allow immune evasion and pathogen distribution. This infectious and especially TB dependent deregulation of the necroptosis/apoptosis pathways ties in with studies reporting upregulation of TRAIL expression in T cells in RA patients compared to controls, correlating with disease activity [64]. This deregulation of cell death and cell cycle pathways may also explain why many cancer-related pathways such as the PI3K/AKT are also enriched in our analysis including GSK3, ITGA, PTEN, and FGF. Deregulation of cell death and enhanced proliferation are features of both cancer and the immune response.

Enrichment in antiviral defense related pathways harboring various deferentially expressed genes such as EIF2AK2, DDX58, OAS1, CASP1, and GSK3B was also seen; these genes have been found to play a role in influenza, HPV, EBV and other viral response-related pathways. Indeed, it has previously been shown in a comparative gene expression profiling study between disease discordant twins with systemic autoimmune diseases including RA that antiviral pathways are consistently upregulated [65]. While the results will ultimately have to be confirmed experimentally, and the links we identify will have to be validated by other studies, we believe that these results provide interesting information on pathways shared by the pathology of two diseases and will help understand their pathogenesis and potential interactions.

## Conclusion

Understanding the causative factors for autoimmune diseases such as RA remains a challenge, especially when studying it in a narrow context like the genetic role or the environmental role alone. The patient’s genetic background (such as the carriage of the HLA-DRB1*04 epitope cluster) is likely to play a role in predisposing patients to be more sensitive towards environmental insults such as infections [45]. Our study could provide further evidence of a role of TB infection in the initiation of autoimmune response in RA, and elucidate possible regulatory mechanisms of this process, by the recognition of Mtb PAMP through PRRs (for instance TLRs), enhancing the production of proinflammatory cytokines (such as IL-17), which promote synovitis, and help in T and B cell activation. This activation will impact on the pathogenicity of the disease through enhanced antigen presentation and cytokine production, by promoting angiogenesis, inflammatory cell infiltration and osteoclast formation. Such processes will eventually cause synovial inflammation and articular damage. The findings reported here will have implications on understanding the pathogenesis of RA in response to various environmental and infectious stimuli. Identified genes may represent new potential targets for the understanding and treatment of patients with comorbidity of RA and TB.

## Supporting information

S1 Fig Datasets meta-analysis pipeline. (PDF)

S2 Fig PCA plots of included datasets gene expression before and after normalization. (PDF)

S3 Fig PCA plot of gene expression data. (PDF)

S4 Fig Quantile-Quantile plot of the Cochran’s Q test. (PDF)

S5 Fig Transcription factor analysis. (PDF)

S1 File PRISMA flow diagram. (PDF)

S1 Table Summary of the datasets integrated in the meta-analysis pipeline. (PDF)

S2 Table List of included samples in the meta-analysis. (PDF)

S3 Table The 50 most highly up- or downregulated genes. (PDF)

S4 Table A list of the significantly deregulated genes. (PDF)
