# Supplementary material for: Gene expression profiling meta-analysis reveals novel gene signatures and pathways shared between tuberculosis and rheumatoid arthritis
Source: PLoS One. 2019 Mar 7;14(3):e0213470. doi: 10.1371/journal.pone.0213470 (PMC6405138; doi:10.1371/journal.pone.0213470)

S1 Fig. Datasets meta-analysis pipeline.

Overview of the selection criteria and data analysis pipeline used. Expression profiles of human whole blood or blood cell populations from case control studies for Tuberculosis (TB) and rheumatoid arthritis (RA) were aquired through the Geoquery package in R, and further data normalization, analysis, and initial visualization were carried out through the different analysis modules in the NetworkAnalyst tool.

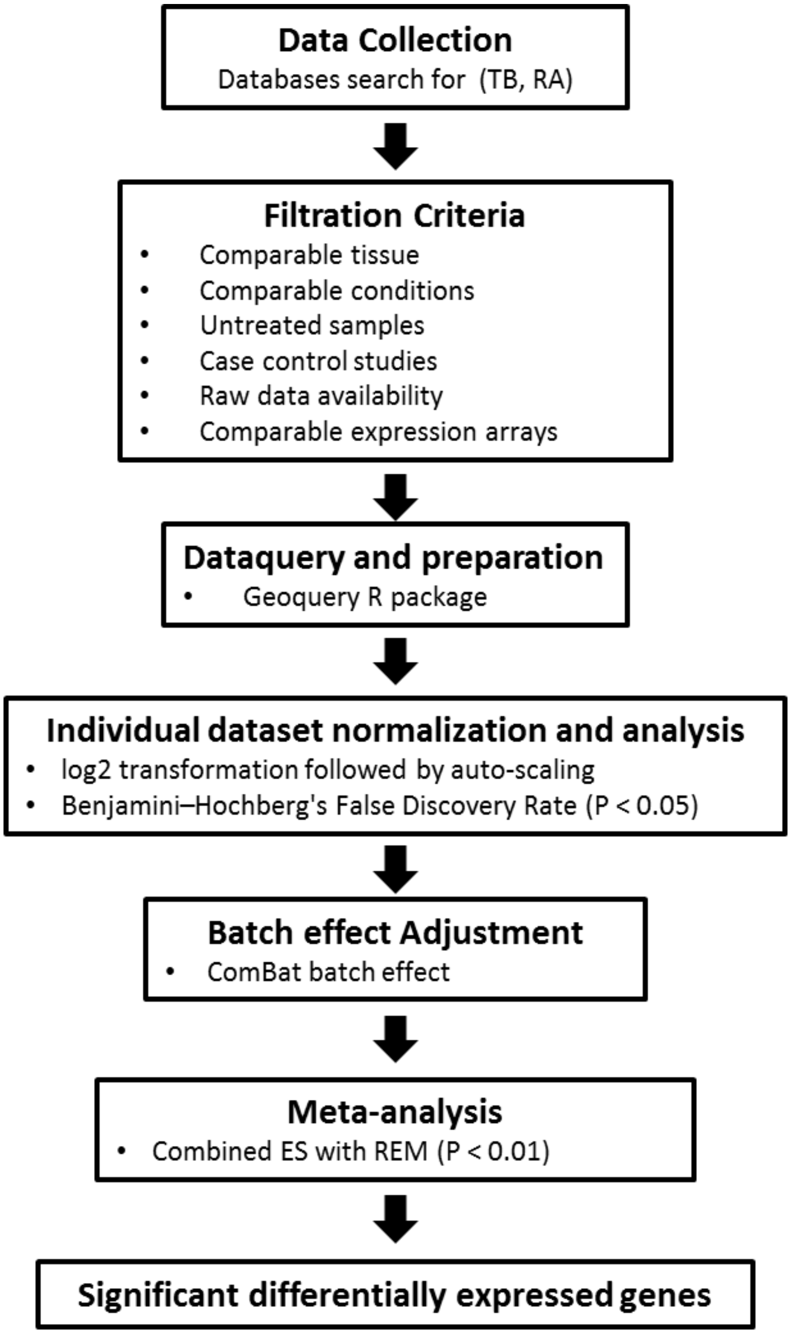

Supplement: S1 Fig — (PDF) [file pone.0213470.s001.pdf]
