# Supplementary material for: Gene expression profiling meta-analysis reveals novel gene signatures and pathways shared between tuberculosis and rheumatoid arthritis
Source: PLoS One. 2019 Mar 7;14(3):e0213470. doi: 10.1371/journal.pone.0213470 (PMC6405138; doi:10.1371/journal.pone.0213470)
Supplement: S2 Fig — (PDF) [file pone.0213470.s002.pdf]

A.

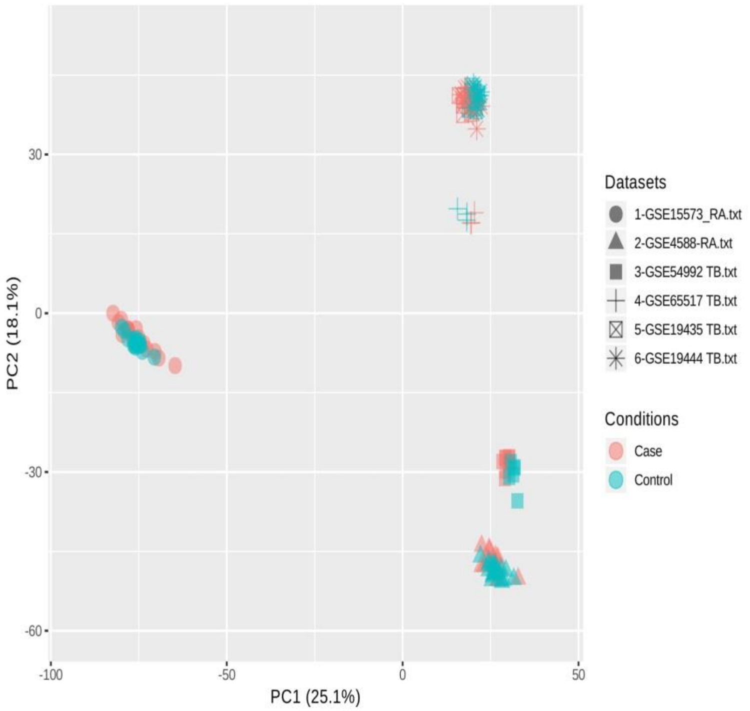

B.

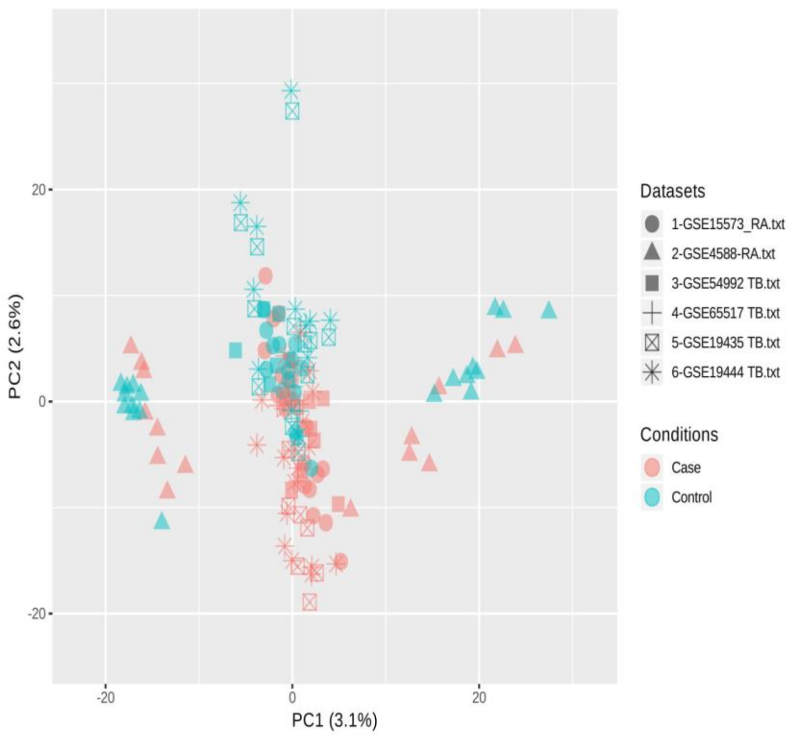

S2 Fig. PCA plots of included datasets gene expression before and after normalization.

The PCA plots of the 6 datasets included in the meta-analysis before (a) and after batch effect adjustment (b). Batch effect has been adjusted using the ComBat batch effect method. The PCA plots are shown based on spanning of their first two principal components. After batch effect adjustment a minimal contribution (3.1%, and 2.6%) can be attributed to the dataset differences, which leads to a minimal contribution of the different batches' variance in the meta-analysis.
