# Supplementary material for: Gene expression profiling meta-analysis reveals novel gene signatures and pathways shared between tuberculosis and rheumatoid arthritis
Source: PLoS One. 2019 Mar 7;14(3):e0213470. doi: 10.1371/journal.pone.0213470 (PMC6405138; doi:10.1371/journal.pone.0213470)
Supplement: S3 Fig — (PDF) [file pone.0213470.s003.pdf]

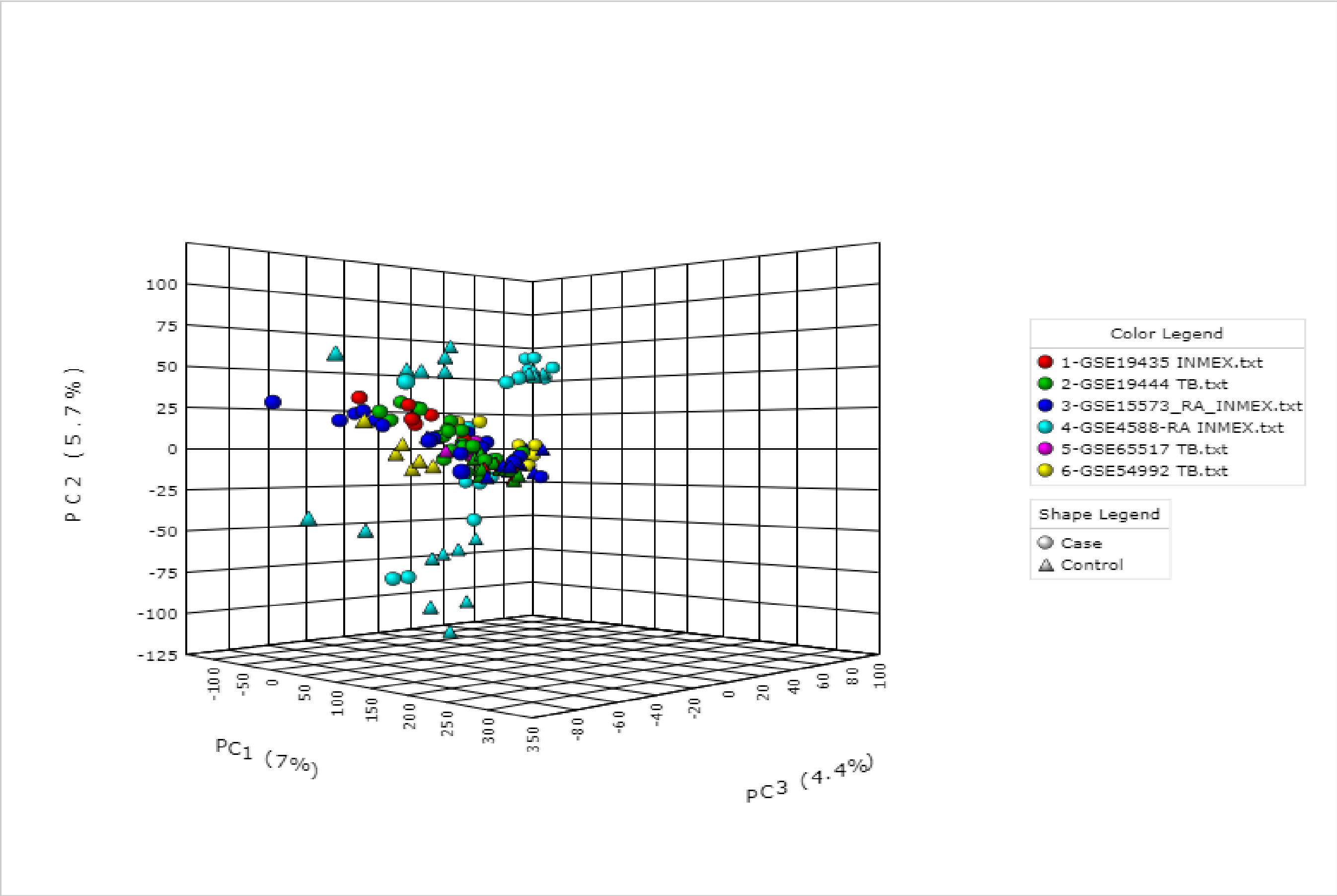

S3 Fig. PCA plot of gene expression data.

The PCA plot of the 6 datasets in the 3D plane is shown based on spanning of their first three principal components. The high span of the second component can be explained by separation based on the different datasets, where a minimal contribution (5.7%) can be attributed to the dataset differences. In general, no major difference that can be attributed to various dataset platforms or conditions that can lead to bias is seen.
