# Supplementary material for: Gene expression profiling meta-analysis reveals novel gene signatures and pathways shared between tuberculosis and rheumatoid arthritis
Source: PLoS One. 2019 Mar 7;14(3):e0213470. doi: 10.1371/journal.pone.0213470 (PMC6405138; doi:10.1371/journal.pone.0213470)
Supplement: S4 Fig — (PDF) [file pone.0213470.s004.pdf]

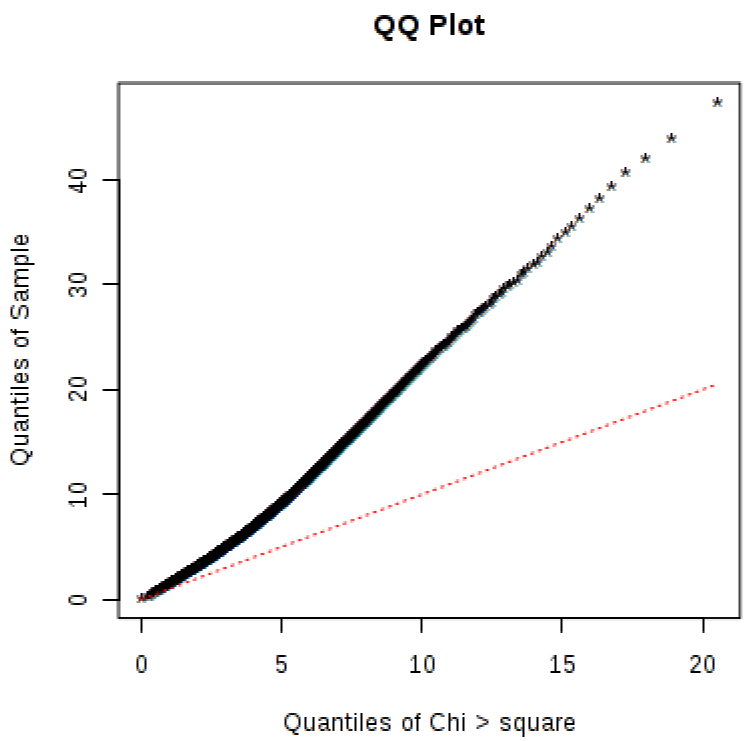

S4 Fig. Quantile-Quantile plot of the Cochran's Q test.

The Quantile-Quantile plot of the Cochran's Q test shows deviation of the Q values from the chi-squared distribution. Based on the Cochran's Q test graphs implementation of the Random Effect model was the appropriate model to analyze the included datasets in the meta-analysis.
