# Supplementary material for: Gene expression profiling meta-analysis reveals novel gene signatures and pathways shared between tuberculosis and rheumatoid arthritis
Source: PLoS One. 2019 Mar 7;14(3):e0213470. doi: 10.1371/journal.pone.0213470 (PMC6405138; doi:10.1371/journal.pone.0213470)
Supplement: S5 Fig — (PDF) [file pone.0213470.s005.pdf]

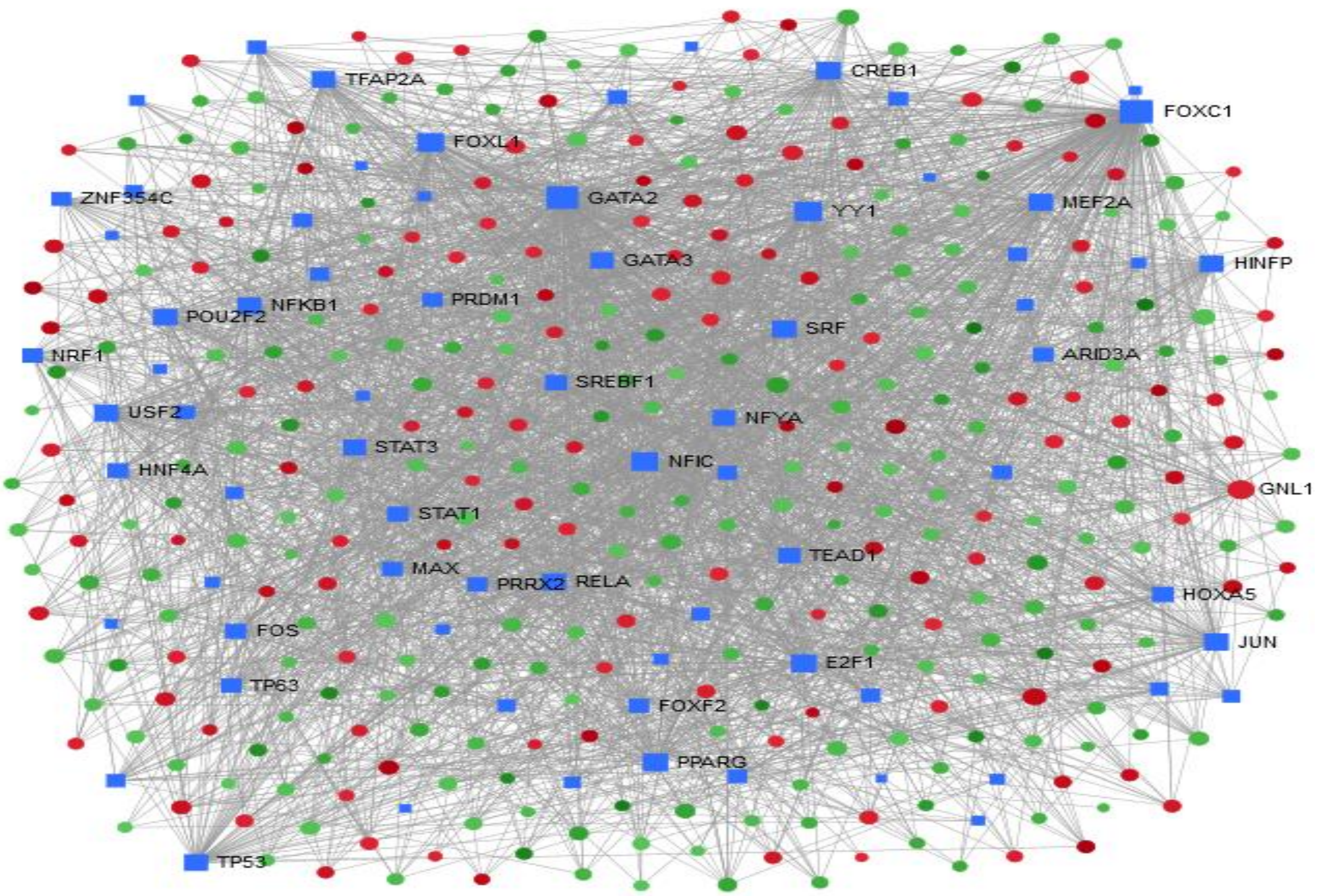

S5 Fig. Transcription factor analysis.

Overrepresented transcription factor binding sites from deferentially expressed genes identified as co-regulated in the samples from patients with TB or RA versus controls are represented using the Transcription factor exploration module in Networkanalyst. JASPAR and ENCODE databases were implemented to scan all the core vertebrate TF binding profiles to identify the TFBS.
