# Supplementary material for: Gene expression profiling meta-analysis reveals novel gene signatures and pathways shared between tuberculosis and rheumatoid arthritis
Source: PLoS One. 2019 Mar 7;14(3):e0213470. doi: 10.1371/journal.pone.0213470 (PMC6405138; doi:10.1371/journal.pone.0213470)
Supplement: S1 Table — (PDF) [file pone.0213470.s007.pdf]

S1 Table. Summary of the datasets integrated in the meta-analysis pipeline.

Six datasets matched our meta-analysis inclusion criteria and were considered for downstream analysis.

A total of 141 samples (41 patients with active TB, 33 RA patients and 67 healthy controls) were included.

RA	PMID	Data set	Platform	Study	cell type	samples	patients	controls	Refrence
1	19710928	GSE15573	GPL6102	Immunity and Defense Genes in Peripheral Blood Mononuclear Cells of Rheumatoid Arthritis patients	PBMC	33	18	15	24
2	NA	GSE4588	GPL570	Identification of genes specifically over-expressed in lupus CD4 T and B cells	CD4 T and B cells	34	15	19	NA
**TB**	**PMID**	**Data set**	**Platform**	**Study**	**cell type**	**samples**	**patients**	**controls**	**Refrence**
1	24647646	GSE54992	GPL570	Increased complement C1q level marks active disease in human tuberculosis	PBMC	15	9	6	25
2	25992611	GSE65517	GPL10558	Comparative analysis of monocytes from healthy donors, patients with metastatic breast cancer, sepsis or tuberculosis	PBMC	6	3	3	26
3	20725040	GSE19435	GPL6947	Transcriptional profiles in Blood of patients with Tuberculosis - Longitudinal Study	Whole blood	19	7	12	27
4	20725040	GSE19444	GPL6947	Blood Transcriptional Profiles of Active and Latent TB	Whole blood	34	22	12	27
