# Supplementary material for: Gene expression profiling meta-analysis reveals novel gene signatures and pathways shared between tuberculosis and rheumatoid arthritis
Source: PLoS One. 2019 Mar 7;14(3):e0213470. doi: 10.1371/journal.pone.0213470 (PMC6405138; doi:10.1371/journal.pone.0213470)
Supplement: S2 Table — (PDF) [file pone.0213470.s008.pdf]

S2 Table. List of included Samples in the meta-analysis.

List of GEO sample accession IDs for the 141 samples included in the meta-analysis. (41 patients with active TB, 33 RA patients, and 67 healthy controls)

Sample ID	Condition	Sample ID	Condition	Sample ID	Condition	Sample ID	Condition
GSM389705	Healthy	GSM484368	Healthy	GSM389703	RA	GSM1327526	TB
GSM389707	Healthy	GSM484369	Healthy	GSM389704	RA	GSM1327527	TB
GSM389709	Healthy	GSM484370	Healthy	GSM389706	RA	GSM1327528	TB
GSM389710	Healthy	GSM484371	Healthy	GSM389708	RA	GSM1327529	TB
GSM389712	Healthy	GSM484372	Healthy	GSM389711	RA	GSM1327530	TB
GSM389713	Healthy	GSM484373	Healthy	GSM389714	RA	GSM1327531	TB
GSM389715	Healthy	GSM484374	Healthy	GSM389716	RA	GSM1327533	TB
GSM389718	Healthy	GSM484375	Healthy	GSM389717	RA	GSM1327535	TB
GSM389720	Healthy	GSM484376	Healthy	GSM389719	RA	GSM1327540	TB
GSM389723	Healthy	GSM484377	Healthy	GSM389721	RA	GSM1599187	TB
GSM389725	Healthy	GSM484378	Healthy	GSM389722	RA	GSM1599188	TB
GSM389728	Healthy	GSM484379	Healthy	GSM389724	RA	GSM1599189	TB
GSM389729	Healthy	GSM484644	Healthy	GSM389726	RA	GSM484380	TB
GSM389732	Healthy	GSM484603	Healthy	GSM389727	RA	GSM484383	TB
GSM389734	Healthy	GSM484604	Healthy	GSM389730	RA	GSM484386	TB
GSM101869	Healthy	GSM484612	Healthy	GSM389731	RA	GSM484387	TB
GSM101870	Healthy	GSM484613	Healthy	GSM389733	RA	GSM484388	TB
GSM101871	Healthy	GSM484622	Healthy	GSM389735	RA	GSM484395	TB
GSM101872	Healthy	GSM484623	Healthy	GSM101879	RA	GSM484398	TB
GSM101873	Healthy	GSM484632	Healthy	GSM101880	RA	GSM484595	TB
GSM101874	Healthy	GSM484633	Healthy	GSM101881	RA	GSM484596	TB
GSM101875	Healthy	GSM484634	Healthy	GSM101882	RA	GSM484597	TB
GSM101876	Healthy	GSM484647	Healthy	GSM101883	RA	GSM484598	TB
GSM101877	Healthy	GSM484648	Healthy	GSM101884	RA	GSM484605	TB
GSM101878	Healthy	GSM1327542	Healthy	GSM101885	RA	GSM484606	TB
GSM101962	Healthy	GSM1327546	Healthy	GSM101886	RA	GSM484610	TB
GSM101963	Healthy	GSM1327544	Healthy	GSM102703	RA	GSM484611	TB
GSM101964	Healthy	GSM1327549	Healthy	GSM102704	RA	GSM484614	TB
GSM101965	Healthy	GSM1327550	Healthy	GSM102709	RA	GSM484618	TB
GSM101966	Healthy	GSM1599181	Healthy	GSM102705	RA	GSM484619	TB
GSM101967	Healthy	GSM1599182	Healthy	GSM484630	TB	GSM484620	TB
GSM101968	Healthy	GSM1599183	Healthy	GSM484631	TB	GSM484621	TB
GSM101969	Healthy	GSM102706	RA	GSM484638	TB	GSM484628	TB
GSM101970	Healthy	GSM102707	RA	GSM484639	TB	GSM484629	TB
GSM1327541	Healthy	GSM102708	RA	GSM484641	TB	GSM484642	TB
						GSM484645	TB
