# Supplementary material for: Gene expression profiling meta-analysis reveals novel gene signatures and pathways shared between tuberculosis and rheumatoid arthritis
Source: PLoS One. 2019 Mar 7;14(3):e0213470. doi: 10.1371/journal.pone.0213470 (PMC6405138; doi:10.1371/journal.pone.0213470)
Supplement: S3 Table — (PDF) [file pone.0213470.s009.pdf]

S3 Table. The 50 most highly up- or downregulated genes.

A list of most significantly differentially regulated genes common to TB and RA vs. controls, detected through the random effect model (REM) in NetworkAnalyst. DEGs were first detected in their respective studies through Benjamini–Hochberg's False Discovery Rate (FDR) with cut-off p-values of < 0.05, before running the REM analysis. In total 341 genes (table S4) were found as significantly regulated by this method (P-value < 0.01).

Upregulated genes:

EntrezID	Name	CombinedES	P-value	EntrezID	Name	CombinedES	P-value
7100	TLR5	1,4674	2,47E-09	22921	MSRB2	1,4496	8,90E-05
8743	TNFSF10	2,0036	4,86E-09	1462	VCAN	0,95884	9,24E-05
55288	RHOT1	1,3542	2,39E-08	5728	PTEN	0,95704	9,24E-05
23765	IL17RA	1,2507	2,43E-07	91056	AP5B1	0,97018	0,00010652
3240	HP	1,1996	9,63E-07	24138	IFIT5	0,94647	0,0001096
22849	CPEB3	1,1736	1,47E-06	64397	ZFP106	1,2214	0,00012596
115207	KCTD12	1,1466	3,41E-06	3142	HLX	0,94776	0,00012676
3176	HNMT	1,1162	5,14E-06	22885	ABLIM3	0,93706	0,00013773
5058	PAK1	1,1049	6,33E-06	10673	TNFSF13B	1,3985	0,00014295
79932	KIAA0319L	1,0984	7,19E-06	2932	GSK3B	0,93698	0,00014441
3934	LCN2	1,0534	1,65E-05	1955	MEGF9	1,5463	0,00014668
8819	SAP30	1,0331	2,97E-05	1903	S1PR3	0,93718	0,00014668
2745	GLRX	1,9062	3,35E-05	10613	ERLIN1	0,93537	0,00014668
79772	MCTP1	1,0588	3,50E-05	10981	RAB32	1,3899	0,00016729
26873	OPLAH	1,1551	4,08E-05	2529	FUT7	1,1976	0,00019552
23643	LY96	1,9692	4,16E-05	1845	DUSP3	1,4934	0,00021618
838	CASP5	1,0912	4,27E-05	433	ASGR2	1,0971	0,00021618
10261	IGSF6	0,9927	5,55E-05	57616	TSHZ3	1,3809	0,000222
8291	DYSF	1,3642	6,43E-05	8402	SLC25A11	0,90323	0,00024482
338339	CLEC4D	1,2795	6,43E-05	9332	CD163	0,92146	0,00027386
84316	LSMD1	1,1187	6,56E-05	23586	DDX58	0,89891	0,00030153
114769	CARD16	1,4257	6,90E-05	23380	SRGAP2	0,89742	0,00031664
10419	PRMT5	0,97711	6,96E-05	1861	TOR1A	0,88923	0,00033299
51065	RPS27L	1,2664	7,61E-05	23569	PADI4	0,88542	0,00033607
91543	RSAD2	0,98354	8,65E-05	51311	TLR8	1,4764	0,00034778

Down-regulated genes:

EntrezID	Name	CombinedES	P-value	EntrezID	Name	CombinedES	P-value
57062	DDX24	1,294	9,64E-08	1408	CRY2	0,87925	0,00042583
26051	PPP1R16B	1,3586	3,34E-07	6191	RPS4X	0,86739	0,00045462
79868	ALG13	1,1823	1,28E-06	79796	ALG9	1,0251	0,00047214
3187	HNRNPH1	1,4745	4,80E-06	64112	MOAP1	1,0755	0,00052434
57602	USP36	1,5677	5,21E-06	10425	ARIH2	1,1568	0,00052595
55602	CDKN2AIP	1,0963	6,99E-06	7486	WRN	1,0711	0,00055602
56257	MEPCE	1,0558	1,65E-05	147694	ZNF548	0,95848	0,00055602
6477	SIAH1	1,055	1,65E-05	10199	MPHOSPH10	0,85553	0,00059339
11044	PAPD7	1,0246	3,27E-05	8665	EIF3F	0,84643	0,00063299
338657	CCDC84	1,1973	3,47E-05	56941	C3orf37	0,87353	0,00063972
65108	MARCKSL1	1,0621	3,61E-05	2254	FGF9	0,84844	0,00066006
54476	RNF216	1,4547	4,20E-05	6259	RYK	0,92941	0,00077098
55683	KANSL3	1,1817	6,43E-05	9117	SEC22C	0,84337	0,00077098
51277	DNAJC27	0,97476	6,90E-05	55846	ITFG2	0,84182	0,00077098
4209	MEF2D	1,0888	7,61E-05	23020	SNRNP200	1,0772	0,00080654
93058	COQ10A	1,1886	9,24E-05	3399	ID3	1,5832	0,00089329
8731	RNMT	1,156	9,24E-05	9267	CYTH1	0,83255	0,00090035
51654	CDK5RAP1	1,1401	0,00012596	2794	GNL1	0,82794	0,00096285
6832	SUPV3L1	1,3574	0,00014449	55661	DDX27	0,83237	0,00096671
7050	TGIF1	1,2326	0,00014668	55274	PHF10	0,82724	0,00096671
79156	PLEKHF1	0,93213	0,00014757	2889	RAPGEF1	0,95124	0,0010094
64745	METTL17	0,9622	0,0001492	23234	DNAJC9	0,82806	0,0010458
23119	HIC2	0,91973	0,00016827	130074	FAM168B	0,81238	0,0013477
9533	POLR1C	1,3076	0,00023909	51569	UFM1	0,80466	0,0013505
113791	PIK3IP1	1,597	0,00042583	54946	SLC41A3	1,0231	0,00144
