# Supplementary material for: Gene expression profiling meta-analysis reveals novel gene signatures and pathways shared between tuberculosis and rheumatoid arthritis
Source: PLoS One. 2019 Mar 7;14(3):e0213470. doi: 10.1371/journal.pone.0213470 (PMC6405138; doi:10.1371/journal.pone.0213470)
Supplement: S4 Table — (PDF) [file pone.0213470.s010.pdf]

S4 Table. A list of the significantly deregulated genes.

List of the significant 341 genes (P-value < 0.01), that was used for pathway enrichment analysis.

Gene official symbol							
DDX24	KANSL2	TLR5	TCIRG1	BATF	RAPGEF1	DDX58	NDUFAF3
PPP1R16B	RNF125	TNFSF10	F13A1	MXD3	DNAJC9	SRGAP2	C22orf39
ALG13	RPL19	RHOT1	S100A12	MFSD1	FAM168B	TOR1A	CLEC1B
HNRNPH1	FBXW4	IL17RA	NIPSNAP3A	TRIB1	UFM1	PADI4	MKNK1
USP36	FAM43A	HP	EXOC6	PRDX4	SLC41A3	TLR8	LAMTOR2
CDKN2AIP	RRP8	CPEB3	PROS1	COX15	PARP6	MLKL	CECR6
MEPCE	WDR74	KCTD12	PLBD1	CTSS	RIOK1	IFIT3	LPAR6
SIAH1	TUBGCP5	HNMT	FBXL5	CMPK2	JOSD1	ITGA2B	CETP
PAPD7	YTHDC1	PAK1	TNFAIP8L2	TMX1	ILKAP	OSTM1	APOBR
CCDC84	ZSCAN18	KIAA0319L	TMEM165	CDKN2C	BSDC1	TMEM8A	SAMD9L
MARCKSL1	EXOSC6	LCN2	F5	VPS29	RALGAPA1	CCDC53	LSM10
RNF216	HNRNPA0	SAP30	YIPF1	COMMD10	BRWD1	SPTLC2	SORT1
KANSL3	LUC7L	GLRX	DHRS7B	FBN2	SAAL1	SIGLEC5	COMMD9
DNAJC27	REV1	MCTP1	RENBP	CFP	DHX9	RNF31	LTBR
MEF2D	RABGGTB	OPLAH	C1QB	SIRPB1	ANKRA2	OAS3	IFI44L
COQ10A	LY9	LY96	MGST1	HAL	CAMK2N1	RTN3	DAD1
RNMT	POLE3	CASP5	B3GNT8	NRD1	PARP15	HOOK3	RNASE2
CDK5RAP1	TYW3	IGSF6	MX2	RGS19	CCR7	STAB1	PARP9
SUPV3L1	AFG3L2	DYSF	BLVRA	ACOT13	GPR183	STOM	KAL1
TGIF1	LANCL2	CLEC4D	C9orf66	MSRB1	WDR19	AURKB	IFI44
PLEKHF1	RPLP0	LSMD1	FKBP15	BRK1	SGSM2	EIF2AK2	STX3
METTL17	STARD5	CARD16	GIMAP2	FAM111A	CYP2E1	IFIT1	SCO2
HIC2	NCBP2	PRMT5	NAGK	MCTS1	PRPS1	NUDT16	LGALS9
POLR1C	SURF6	RPS27L	MKL1	KIF15	CLEC7A	CRISPLD2	LDB3
PIK3IP1	S1PR1	RSAD2	IGFL2	TK2	ATXN7L2	IGF2BP3	P2RY10
CRY2	PTBP2	MSRB2	ATG7	NUDT22	RINT1	SLC22A16	HK3
RPS4X	DMTF1	VCAN	FOLR3	ECHDC3	BIN1	RNF169	FGF9
ALG9	GABPA	PTEN	C2orf88	TMBIM4	MCOLN2	CASP1	RYK
MOAP1	PASK	AP5B1	ANKRD22	C4orf34	DUSP2	KIAA0040	SEC22C
ARIH2	E4F1	IFIT5	S100A6	DHTKD1	EIF3L	VWA5A	ITFG2
WRN	SNRPA1	ZFP106	TMLHE	SHKBP1	THOC1	TREML1	SNRNP200
ZNF548	SELM	HLX	MPO	CSTA	ELP2	THOC5	ID3
MPHOSPH10	RPL37	ABLIM3	ANKRD35	ILK	SHISA2	DBI	CYTH1
EIF3F	WDR43	TNFSF13B	SASH3	SCCPDH	RPS19	ATP6V0E1	GNL1
C3orf37	MAP3K4	GSK3B	DSC2	MARCO	LONP1	PML	DDX27
PHF10	CXorf40A	MEGF9	PIGF	SH3BGRL2	PVRIG	PRM3	ARHGEF7
ZBED4	DNAJC18	S1PR3	LMO2	PARP10	MED10	TRAFD1	FOSB
PRNP	RUNX3	ERLIN1	PSMA4	NAGA	TRABD	SKA2	POLR2D
FLYWCH1	NUP107	RAB32	KLHL8	FAM198B	ZNF212	C1QA	LST1
TSHZ3	IQGAP1	FUT7	EIF2C4	IFI27	LRP5L	DUSP7	VAMP5
SLC25A11	TMEM180	DUSP3	POLE4	XAF1	FN3KRP	ACOX1	MYL12A
CD163	VAMP8	ASGR2	LIN7A	LRP1	LAS1L	NEXN	NDUFS4
TOMM20	AGPAT1	EEF2	MTIF3	PRPF8			
